# Intra-cardiac MR imaging & MR-tracking catheter for improved MR-guided EP

**DOI:** 10.1186/1532-429X-17-S1-P237

**Published:** 2015-02-03

**Authors:** Yue Chen, Zion T Tse, Wei Wang, Raymond Y Kwong, William G Stevenson, Ehud J Schmidt

**Affiliations:** Engineering, The University of Georgia, Athens, GA USA; Radiology, Brigham and Women’s Hospital, Boston, MA USA; Cardiology, Brigham and Women’s Hospital, Boston, MA USA

## Background

Electrophysiology (EP) studies can diagnose & treat patients with arrhythmia. MR-guided EP is growing, driven by the ability of cardiac MRI to provide high-contrast images. For intra-procedural use, MRI provides images of the acute state of radio-frequency ablation (RFA) lesions, e.g. necrosis, edema and hemorrhage, that potentially reduce recurrences & complications [1, 2]. Unfortunately, acquisition of these images using surface MRI coils, considering the high-spatial-resolution (~1x1x2mm^3^) requirements, can be lengthy (scar~10 mins/scan, edema~12 mins/scan) [3], severely increasing the duration of MR-guided procedures. As demonstrated in other body regions, e.g. endorectal MRI, and with other imaging modalities (Intra-Cardiac Echo), intracavitary probes provide increased Signal-to-Noise-Ratio (SNR), due to their proximity to the area of interest. An Intra-cardiac MR imaging (ICMRI) coil may provide substantially higher SNR, but a complete application must also provide accurate heart motion compensation [4], in order to produce non-blurred images. We constructed an ICMRI catheter, with integrated imaging & positional-tracking elements, optimized for (1) cardiovascular introduction as a sheath "riding on" an EP ablation catheter & for (2) close-proximity imaging (~4 cm FOV) during RFA delivery.

## Methods

**Figure 1:**
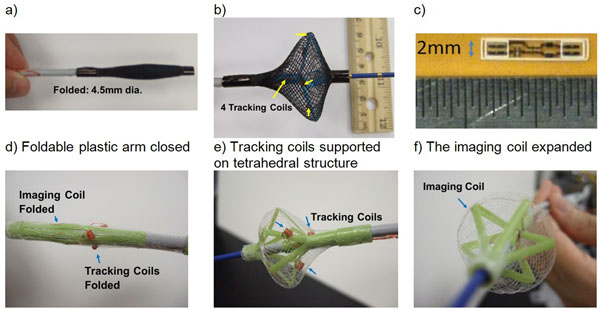
ICMRI catheter with imaging coil (a) collapsed to allow navigation within the vasculature, and (b) expanded for imaging; (c) tuning/matching micro-electronics. ICMRI without protective nylon mesh, showing the foldable plastic arm structure, on which both the imaging and tracking coils were mounted, during closing (d) and opening (e-f).

The ICMRI catheter consists of a deployable imaging coil & 4 tracking micro-coils at the catheter tip. The imaging coil is folded during vascular navigation (4.5-mm diameter). During the expansion, the imaging coil forms a circular loop of 40mm in diameter which images a ~4cm FOV, while the tracking coils form a tetrahedral array (Fig. 1a-b, d-f) for accurate motion-compensation [4]. The imaging coil, constructed of two windings of 38-gauge copper wire, was woven into an expandable protective nylon mesh. The foldable plastic arms (Fig. 1d), covered by the nylon mesh, provide a tetrahedral structure on which the 4 tracking micro-coils (2mm diameter) were mounted. The imaging coil was connected to a 123 MHz (3T) miniaturized tuning/matching circuit in a pocket on the ICMRI sheath (Fig. 1c) (~11 dB reflection-coefficient, loaded). To evaluate imaging performance, the ICMRI was compared to a 32-channel Invivo cardiac-array during ex-vivo swine left-ventricular (LV) & left-atrial (LA) imaging.

## Results

**Figure 2:**
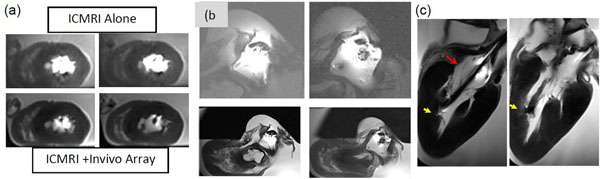
ICMRI imaging at 3T (a) Breath-held T2-w; LV short-axis; ICMRI alone (Top) versus ICMRI+ Invivo array (Bottom), (b) LA short axis; ICMRI alone (Top, 2.1X magnified) versus ICMRI+Invivo array (Bottom, 1X magnified); (c) LV long-axis T2-w. ICMRI catheter (red arrow) mounted on EP ablation catheter, showing the EP catheter tip (yellow arrows) contacting the papillary muscle.

ICMRI provided 2-4 times the SNR of the Invivo array at distances of 5-8 cm from the coil, for both T1-w GRE and T2-w TSE (Fig. 2). Efficient breath-hold (20-sec) T2-w scans were also possible. Operation in tandem with an MR-compatible EP ablation catheter (St. Jude Medical) was also demonstrated.

## Conclusions

The ICMRI catheter allows for 4-16x faster imaging during MR-guided RFA, improving temporal efficiency. ICMRI supports catheterization, in analogy with ICE's advantages (trans-septal puncture, valve passage), supporting low-SNR imaging contrasts (Strain, Diffusion).

## Funding

AHA 10SDG261039, NIH R03 EB013873-01A1, NIH U41-RR019703.
